# Genomic, epigenomic, and immune subtype analysis of CTSL/B and SARS-CoV-2 receptor ACE2 in pan-cancer

**DOI:** 10.18632/aging.104147

**Published:** 2020-11-20

**Authors:** Huimin Li, Longxiang Xie, Lin Chen, Lu Zhang, Yali Han, Zhongyi Yan, Xiangqian Guo

**Affiliations:** 1 Institute of Biomedical Informatics, Cell Signal Transduction Laboratory, Bioinformatics Center, Henan Provincial Engineering Center for Tumor Molecular Medicine, School of Basic Medical Sciences, Henan University, Kaifeng 475004, China; 2 Department of Histology and Embryology, Henan International Joint Laboratory for Nuclear Protein Regulation, School of Basic Medical Sciences, Henan University, Kaifeng 475004, China

**Keywords:** SARS-CoV-2, CTSL/B, COVID-19

## Abstract

SARS-coronavirus 2 (SARS-CoV-2) has been spreading widely and posing an international challenge for both healthcare and society. At present, cancer has been identified as an individual risk factor for COVID-19. Angiotensin converting enzyme 2 (ACE2) and Cathepsin L/Cathepsin B (CTSL/B), which act as the receptor and entry-associated proteases of SARS-CoV-2 respectively, are pivotal for SARS-CoV-2 infection. To investigate the possible SARS-CoV-2 infection risk of pan-cancer, we analyzed the genetic alterations, RNA expression, DNA methylation, and the association with immune subtypes of ACE2 and CTSL/B with the prognosis in pan-cancer. Results showed the upregulation of CTSL/B and ACE2 in Pancreatic adenocarcinoma (PAAD) and Stomach adenocarcinoma (STAD) and demonstrated a positive correlation between copy number alteration (CNA) and gene expression for CTSB in PAAD and STAD. Hypomethylation and a negative correlation of gene expression and methylation for CTSB were detected in PAAD. In addition, ACE2 and CTSL/B are overexpressed in the IFN-gamma immune subtype of ovarian serous Cystadenocarcinoma (OV), Cervical squamous cell carcinoma and endocervical adenocarcinoma (CESC), and Bladder urothelial carcinoma (BLCA). Our study presents a bioinformatics assessment for the potential risk of SARS-CoV-2 infection in pan-cancer.

## To the Editor

The worldwide spread of COVID-19 is posing a global challenge for both healthcare and society. The disease caused by SARS-CoV-2 has symptoms similar to those caused by SARS-CoV and MERS-CoV. To date, more than 10 million people worldwide have been diagnosed with COVID-19, which has caused over 550,000 deaths. Since COVID-19 started to spread in the world, patients with cancer were designated as a particularly vulnerable sub-group of the population [1]. At present, the COVID-19 and Cancer Consortium (CCC19), a consortium of over 120 cancer centers and other organizations that have come together to collect data about patients with cancer who have been diagnosed with COVID-19, included patients with several types of malignancies (gastrointestinal, 12%; breast cancer, 21%; thoracic cancers, 10%; hematological malignancies, 22%; and others, 19%) [1, 2]. SARS-Cov-2 entry into a cell is dependent upon the cellular membrane receptor ACE2 which binds the viral spike (S) protein, and the host cell proteases such as CTSL/B which cleave and activate the viral spike (S) protein. Coronaviruses utilize the viral spike (S) protein to bind to their cellular receptors. Such binding and cleavage of the S protein into two subunits named S1 and S2 by host proteases lead to fusion between the cell and viral membranes and viral activation for viral infection. SARS-CoV-2 uses the ACE2 receptor for entry [3]. The S1/S2 cleavage site of SARS-CoV-2 is between the threonine and methionine at positions 696 and 697, and is identical to that of SARS-CoV which has been shown to be cleaved by CTSL [4]. SARS-CoV takes advantage of the endosomal cysteine proteases CTSL and CTSB. In healthy individuals those organs including lung, heart, kidney, bladder and oesophagus which express a high level of ACE2 seem to be more vulnerable to SARS-CoV-2 infection [5]. Hence, the expression and distribution of ACE2 and CTSL/B may explain the different susceptibility to SARS-Cov-2 infection for pan-cancer. To address this possibility, we performed a comprehensive analysis of CTSL/B and ACE2 expression by using genomic, transcriptomic and epigenomic data of pan-cancer so as to provide a reference for the assessment of SARS-CoV-2 infection in pan-cancer.

**Figure 1:**
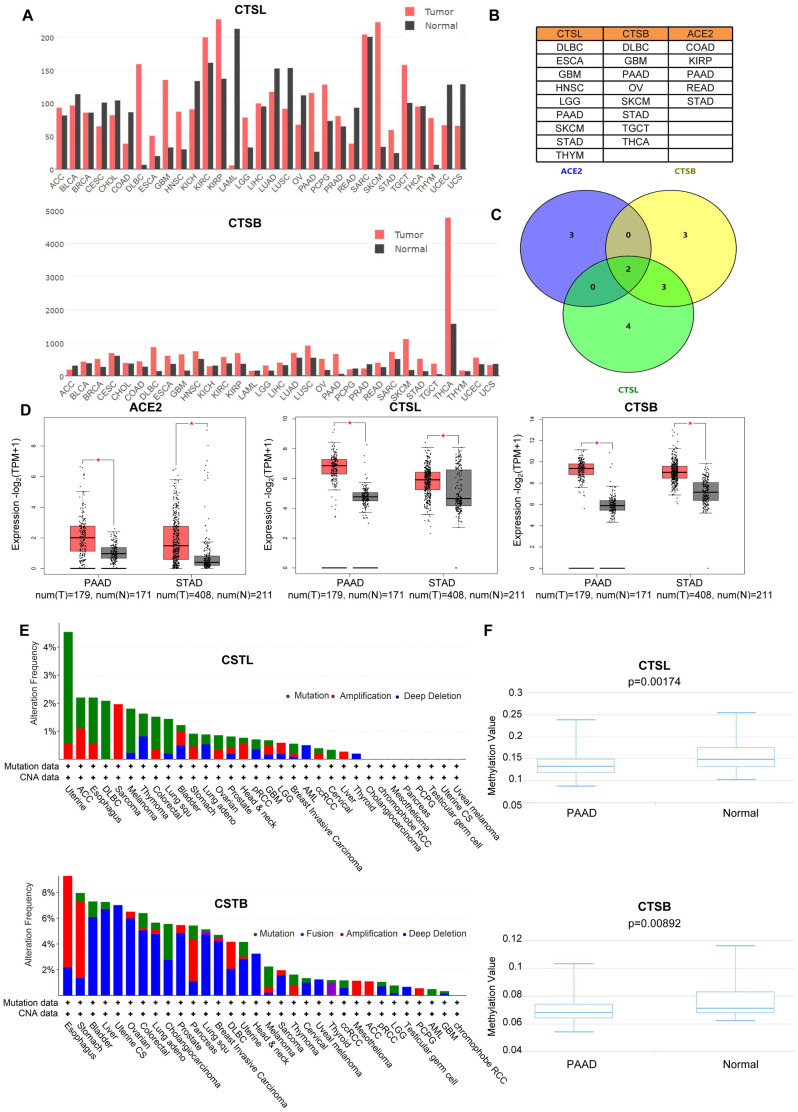
**The analyses of gene variation and epigenetics of CTSL/B and ACE2 in pan-cancer.** (**A**) The distribution of differential expression of CTSL/B in 31 types of cancers and adjacent tissues based on TCGA and Genotype-Tissue Expression (GTEx) data (GEPIA). (**B**) The exhibition of tumors upregulating CTSL/B and ACE2, respectively. (**C**) The overlap of tumors overexpressing ACE2, CTSL/B using venny 2.0.2. (**D**) The significant difference analysis of CTSL/B in PAAD and STAD. (**E**) The mutation and CNA distribution of CTSL/B in pan-cancer (cBioportal). (**F**) The statistical difference graphs of DNA methylation for CTSB/L in PAAD (DiseaseMeth version 2.0). *p*<0.05 was regarded as significant.

Based on the transcriptional level analysis in GEPIA [6], we found an elevation of CTSL in nine types of tumors including Lymphoid Neoplasm Diffuse Large B-cell Lymphoma (DLBC), Esophageal carcinoma (ESCA), Glioblastoma multiforme (GBM), Head and Neck squamous cell carcinoma (HNSC), LGG, PAAD, Skin Cutaneous Melanoma (SKCM), STAD and Thymoma (THYM) (Figure 1A), and an increase in CTSB in DLBC, GBM, OV, PAAD, SKCM, STAD, Testicular Germ Cell Tumors (TGCT) and Thyroid carcinoma (THCA) (Figure 1A, 1B). Notably, ACE2 is upregulated in Colon adenocarcinoma (COAD), Kidney renal papillary cell carcinoma (KIRP), PAAD, Rectum adenocarcinoma (READ) and STAD (Supplementary Figure 1A, 1B). Further analysis revealed that ACE2 and CTSL/B are all highly expressed in PAAD and STAD (Figure 1C, 1D). Oncomine analysis [7] verified the upregulation (*p*<0.05, fold change>1.5) of CTSL/B in PAAD and STAD when compared to normal tissues (Supplementary Tables 1, 2). With respect to ACE2 in the Oncomine analysis, the small sample size likely contributes to the lack of significant over-expression in PAAD and STAD as compared to normal tissues.

CNA, mutation and DNA methylation can prompt the variation of gene expression. CNA includes amplification, gain, diploidy, shallow deletion and deep deletion. c-BioPortal analysis [8] based on The Cancer Genome Atlas (TCGA) data indicates that genomic amplification increased the gene expression of CTSB in STAD and PAAD (Figure 1E and Supplementary Figure 2, R>0.2, *p*<0.05). For CTSL in STAD, its expression could be elevated by genomic amplification as well (Figure 1E). For ACE2, DNA mutation acts as the dominant factor in regulating gene expression in STAD and PAAD (Supplementary Figure 1B). However, the correlation analysis between gene expression and CNA/mutation showed neither ACE2 nor CTSL expression is relevant to mutation or CNA (Supplementary Figures 3, 4). For other types of tumors, the most frequent DNA alteration of the CTSL gene is DNA mutation in Uterine Corpus Endometrial Carcinoma (UCEC), ESCA, DLBC and Melanoma (Figure 1E), and followed by amplification in Sarcoma (SARC), Adrenocortical carcinoma (ACC), UCEC and ESCA. For CTSB, deep deletion in Uterine Carcinosarcoma (UCS), Liver hepatocellular carcinoma (LIHC), BLCA and OV, is the most frequent DNA alteration, followed by amplification in ESCA, STAD, PAAD and DLBC. For ACE2, the most frequent DNA alteration is mutation in UCEC, UCS, STAD and MEL (Supplementary Figure 1B). To explore the influence of epigenetic modification on the mRNA levels for CTSL/B and ACE2 in PAAD and STAD, seven, fourteen and six CpG probes targeting the promoters of the CTSL, CTSB and ACE2 genes, respectively, using Methylation450k profiles were utilized to recapitulate the DNA methylation levels (Supplementary Table 3) in the DiseaseMeth database [9]; the results demonstrated that DNA methylation of CTSL/B is significantly decreased in PAAD but not in STAD when compared with normal tissues (Figure 1F and Supplementary Figure 5). However, the analysis based on DNMIVD [10] showed that gene expression of CTSB rather than CTSL and ACE2 is negatively correlated with DNA methylation in PAAD and STAD (Supplementary Tables 4–6, R<-0.1, *p*<0.05). Together, the analyses suggest that both DNA methylation and CNA could influence CTSB gene expression in PAAD, while only CNA can affect CTSB gene expression in STAD. For proteomic analysis, we analyzed the expression variation of ACE2 and CTSL/CTSB with TCGA proteomics data, which includes measuring the expression of 261 proteins; unfortunately, these three proteins (ACE2, CTSL and CTSB) were not included in the 261 proteins.

**Figure 2:**
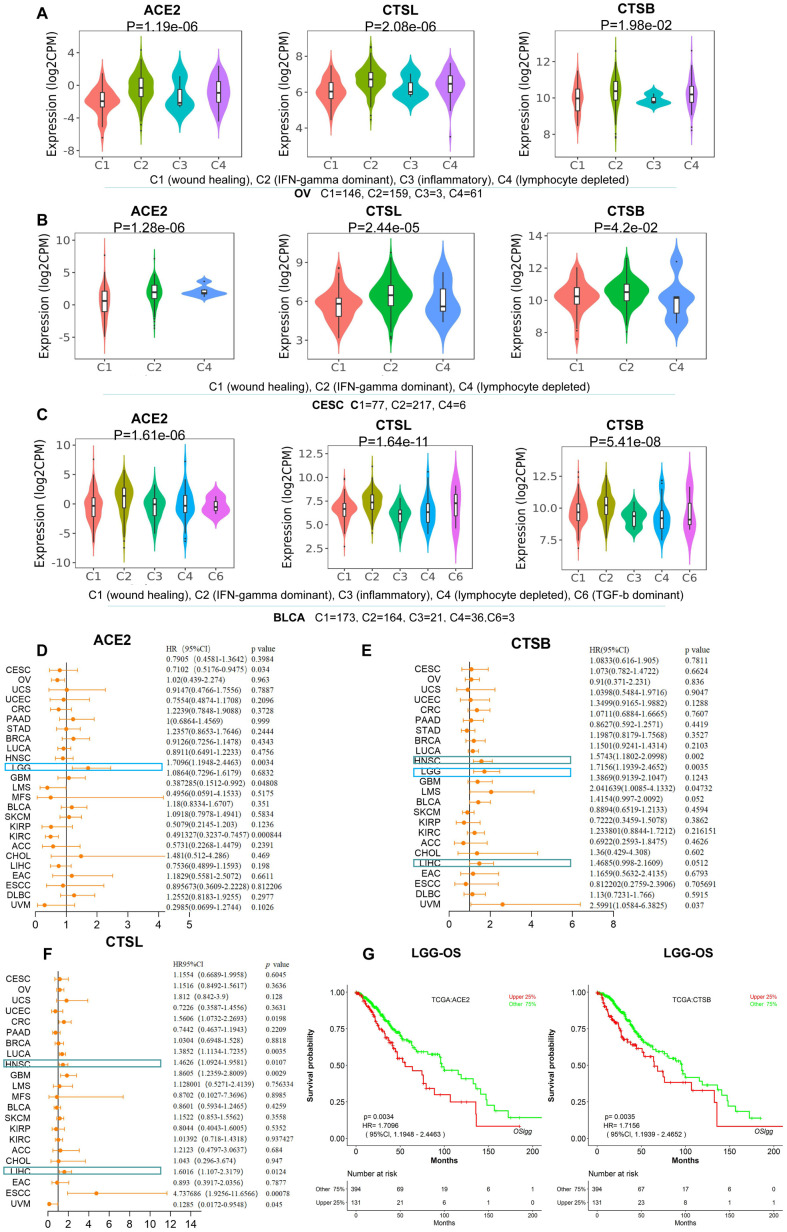
**The identification of immune subtypes and prognosis for CTSL/B and ACE2 in pan-cancer.** (**A**–**C**) The distribution graphs of ACE2 and CTSL/B in six immune subtypes in OV, CESC and BLCA. ACE2 and CTSL/B in OV, CESC and BLCA were most highly expressed in the C2 named IFN-gamma dominant subtype. (**D**–**F**) Forest maps analysis of overall survival for ACE2 and CTSL/B in pan-cancer (LOGpc). The blue boxes represent the overlap of tumors in which ACE2 and CTSB can predict adverse prognosis; the green boxes represent the overlap of tumors in which CTSB and CTSL can predict adverse prognosis. (**G**) The overall survival analyses of ACE2 and CTSB in LGG utilizing the LOGpc online tool based on TCGA data, with results showing that ACE2 and CTSB both predict poor prognosis. *p*<0.05 was regarded as significant; HR>1, *p*<0.05 represented poor prognosis.

We further studied the involvement of ACE2 and CTSL/B in cancer immune subtypes including C1 (wound healing), C2 (IFN-gamma dominant), C3 (inflammatory), C4 (lymphocyte depleted), C5 (immunologically quiet) and C6 (TGF-b dominant) in pan-cancer (Supplementary Figure 6) according to TISIDB [11]. As compared with other types of tumors, BRCA, HNSC, OV, KIRC, CESC and BLCA present greater differential ACE2 expression. Combining the immune subtype analysis of ACE2 and CTSL/B in the six tumor types, we found that ACE2 and CTSL/B are most highly overexpressed in the IFN-gamma dominant immune subtype of OV, CESC and BLCA (Figure 2A–2C), yet not in BRCA, HNSC and KIRC (Supplementary Figure 7). The results indicated that the IFN-gamma dominant immune subtype of OV, CESC and BLCA may provide more opportunities than other immune subtypes for SARS-CoV-2 infection.

To explore the prognostic values of CTSL/B and ACE2, we analyzed gene expression profiling and long-term follow-up data from TCGA using LOGpc online tools and discovered that upregulated CTSL and CTSB both predict poor prognosis in HNSC and LIHC (Supplementary Figure 8). Respectively, CTSL predicts poor overall survival (OS) in six types of tumors including Colorectal cancer (CRC), Lung cancer (LUCA), HNSC, GBM, LIHC and ESCC; overexpression of CTSB predicts unfavorable OS in four types of tumors including HNSC, LGG, Leiomyosarcoma (LMS) and LIHC (Figure 2D–2F). ACE2 predicts poor prognosis only in LGG in which CTSB also portends poor prognosis (Figure 2G). Although the three genes did not concurrently predict poor prognosis in a given type of tumor, the prognostic roles of two of the three pivotal factors for COVID-19 infection may be of value in predicting COVID-19 infection risk in cancers. Hence, LGG, HNSC and LIHC may indicate a greater risk for COVID-19 infection than other types of cancers, but needs further clinical validation. Yu-Jun Dai et al. also emphasized that LIHC patients with high expression level of ACE2 should be more cautious of virus infection. Both studies may provide potential clues for preventing infection of SARS-CoV-2 in cancers [5].

CTSL/B have been reported to be a vital regulators in proliferation, metastasis [12], invasion [13] and prognosis [14] in various types of cancers. For instance, Cathepsins B and L could drive the invasive growth of human melanoma cells [15], and overexpression of CTSL is a marker of invasion and metastasis in ovarian cancer [16], together manifesting a potential capacity for the two proteases CTSL/B as malignant phenotype markers for pan-cancer. In addition, the finding that ACE2 overexpression presents a poor prognosis in LGG, consistent with previous reports [17].

Regarding COVID patients with malignancies, LUAD patients with overexpressed ACE2 were found to have a higher incidence of COVID-19 [18]. Herein, we showed that both ACE2 and CTSL/B are upregulated in PAAD and STAD and are overexpressed in IFN-gamma immune subtypes of OV, CESC and BLCA, implying an infection risk for SARS-CoV-2 in PAAD and STAD, and for IFN-gamma dominance in OV, CESC and BLCA. To clarify these results derived from database analyses, further verification in a large clinical cohort is essential.

## Supplementary Material

Supplementary Figures

Supplementary Tables

## References

[1] Curigliano G. Cancer patients and risk of mortality for COVID-19. Cancer Cell. 2020; 38:161–63. 10.1016/j.ccell.2020.07.006 32710820 PMC7381394

[2] Kuderer NM, Choueiri TK, Shah DP, Shyr Y, Rubinstein SM, Rivera DR, Shete S, Hsu CY, Desai A, de Lima Lopes G Jr, Grivas P, Painter CA, Peters S, et al, and COVID-19 and Cancer Consortium. Clinical impact of COVID-19 on patients with cancer (CCC19): a cohort study. Lancet. 2020; 395:1907–18. 10.1016/S0140-6736(20)31187-9 32473681 PMC7255743

[3] Lan J, Ge J, Yu J, Shan S, Zhou H, Fan S, Zhang Q, Shi X, Wang Q, Zhang L, Wang X. Structure of the SARS-CoV-2 spike receptor-binding domain bound to the ACE2 receptor. Nature. 2020; 581:215–20. 10.1038/s41586-020-2180-5 32225176

[4] Smieszek SP, Przychodzen BP, Polymeropoulos MH. Amantadine disrupts lysosomal gene expression: a hypothesis for COVID19 treatment. Int J Antimicrob Agents. 2020; 55:106004. 10.1016/j.ijantimicag.2020.106004 32361028 PMC7191300

[5] Dai YJ, Hu F, Li H, Huang HY, Wang DW, Liang Y. A profiling analysis on the receptor ACE2 expression reveals the potential risk of different type of cancers vulnerable to SARS-CoV-2 infection. Ann Transl Med. 2020; 8:481. 10.21037/atm.2020.03.61 32395525 PMC7210193

[6] Tang Z, Li C, Kang B, Gao G, Li C, Zhang Z. GEPIA: a web server for cancer and normal gene expression profiling and interactive analyses. Nucleic Acids Res. 2017; 45:W98–102. 10.1093/nar/gkx247 28407145 PMC5570223

[7] Rhodes DR, Yu J, Shanker K, Deshpande N, Varambally R, Ghosh D, Barrette T, Pandey A, Chinnaiyan AM. ONCOMINE: a cancer microarray database and integrated data-mining platform. Neoplasia. 2004; 6:1–6. 10.1016/s1476-5586(04)80047-2 15068665 PMC1635162

[8] Cerami E, Gao J, Dogrusoz U, Gross BE, Sumer SO, Aksoy BA, Jacobsen A, Byrne CJ, Heuer ML, Larsson E, Antipin Y, Reva B, Goldberg AP, et al. The cBio cancer genomics portal: an open platform for exploring multidimensional cancer genomics data. Cancer Discov. 2012; 2:401–04. 10.1158/2159-8290.CD-12-0095 22588877 PMC3956037

[9] Xiong Y, Wei Y, Gu Y, Zhang S, Lyu J, Zhang B, Chen C, Zhu J, Wang Y, Liu H, Zhang Y. DiseaseMeth version 2.0: a major expansion and update of the human disease methylation database. Nucleic Acids Res. 2017; 45:D888–95. 10.1093/nar/gkw1123 27899673 PMC5210584

[10] Ding W, Chen J, Feng G, Chen G, Wu J, Guo Y, Ni X, Shi T. DNMIVD: DNA methylation interactive visualization database. Nucleic Acids Res. 2020; 48:D856–62. 10.1093/nar/gkz830 31598709 PMC6943050

[11] Ru B, Wong CN, Tong Y, Zhong JY, Zhong SS, Wu WC, Chu KC, Wong CY, Lau CY, Chen I, Chan NW, Zhang J. TISIDB: an integrated repository portal for tumor-immune system interactions. Bioinformatics. 2019; 35:4200–02. 10.1093/bioinformatics/btz210 30903160

[12] Liu P, Kong L, Jin H, Wu Y, Tan X, Song B. Differential secretome of pancreatic cancer cells in serum-containing conditioned medium reveals CCT8 as a new biomarker of pancreatic cancer invasion and metastasis. Cancer Cell Int. 2019; 19:262. 10.1186/s12935-019-0980-1 31632196 PMC6788113

[13] Rafn B, Nielsen CF, Andersen SH, Szyniarowski P, Corcelle-Termeau E, Valo E, Fehrenbacher N, Olsen CJ, Daugaard M, Egebjerg C, Bøttzauw T, Kohonen P, Nylandsted J, et al. ErbB2-driven breast cancer cell invasion depends on a complex signaling network activating myeloid zinc finger-1-dependent cathepsin B expression. Mol Cell. 2012; 45:764–76. 10.1016/j.molcel.2012.01.029 22464443

[14] Jain M, Bakhshi S, Shukla AA, Chauhan SS. Cathepsins B and L in peripheral blood mononuclear cells of pediatric acute myeloid leukemia: potential poor prognostic markers. Ann Hematol. 2010; 89:1223–32. 10.1007/s00277-010-1012-3 20567828

[15] Yin M, Soikkeli J, Jahkola T, Virolainen S, Saksela O, Hölttä E. TGF-β signaling, activated stromal fibroblasts, and cysteine cathepsins B and L drive the invasive growth of human melanoma cells. Am J Pathol. 2012; 181:2202–16. 10.1016/j.ajpath.2012.08.027 23063511

[16] Sui H, Shi C, Yan Z, Wu M. Overexpression of Cathepsin L is associated with chemoresistance and invasion of epithelial ovarian cancer. Oncotarget. 2016; 7:45995–6001. 10.18632/oncotarget.10276 27351223 PMC5216776

[17] Chai P, Yu J, Ge S, Jia R, Fan X. Genetic alteration, RNA expression, and DNA methylation profiling of coronavirus disease 2019 (COVID-19) receptor ACE2 in Malignancies: a pan-cancer analysis. J Hematol Oncol. 2020; 13:43. 10.1186/s13045-020-00883-5 32366279 PMC7197362

[18] Kong Q, Xiang Z, Wu Y, Gu Y, Guo J, Geng F. Analysis of the susceptibility of lung cancer patients to SARS-CoV-2 infection. Mol Cancer. 2020; 19:80. 10.1186/s12943-020-01209-2 32345328 PMC7186321

