# Supplementary material for: Genomic, epigenomic, and immune subtype analysis of CTSL/B and SARS-CoV-2 receptor ACE2 in pan-cancer
Source: Aging (Albany NY). 2020 Nov 20;12(22):22370–89. doi: 10.18632/aging.104147 (PMC7746364; doi:10.18632/aging.104147)
Supplement: Supplementary Figures [file aging-12-104147-s001..pdf]

SUPPLEMENTARY FIGURES

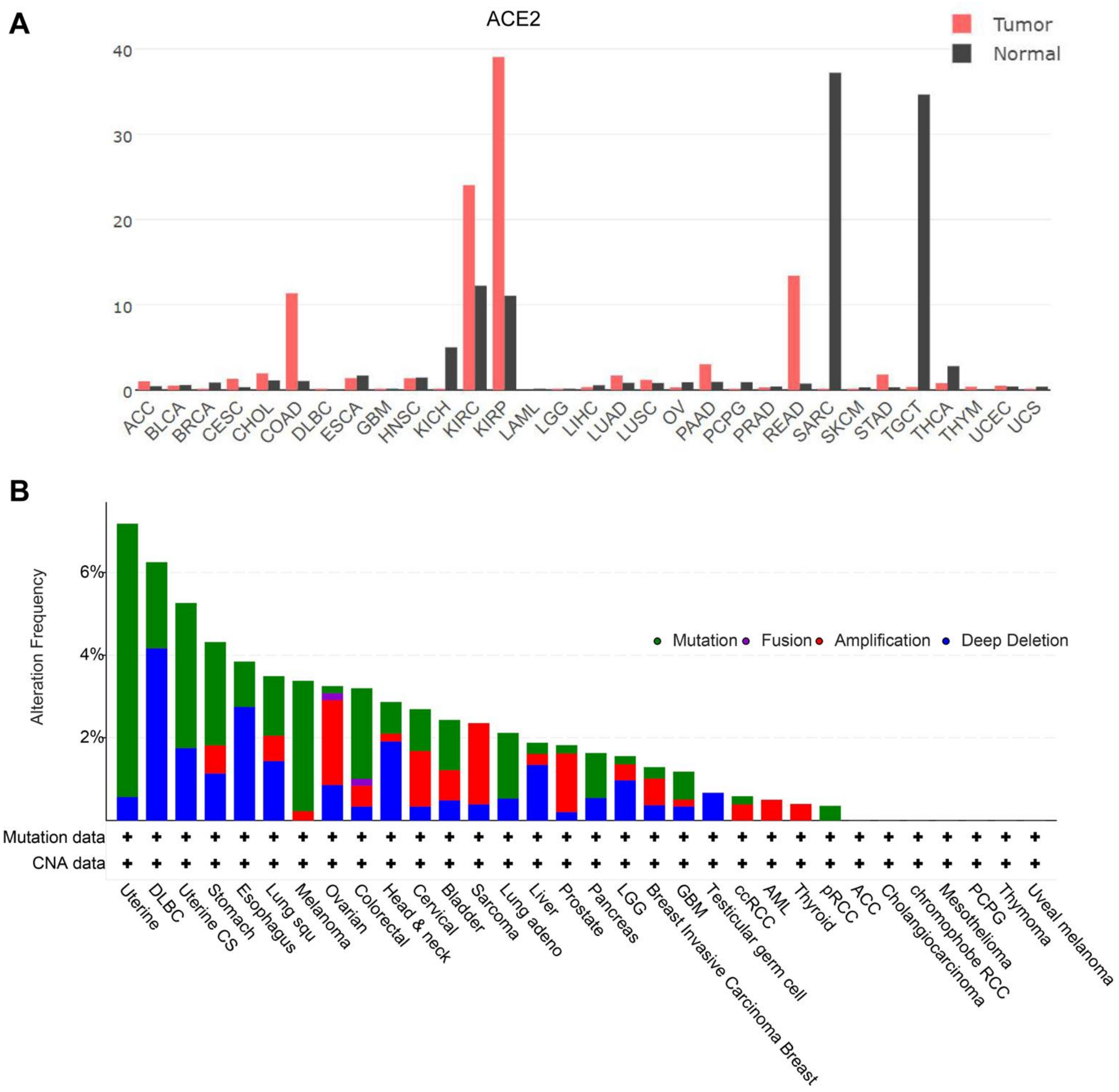

Supplementary Figure 1. (**A**) The distribution of different expression of ACE2 in 31 types of cancers and adjacent normal based on TCGA and GTEx data (GEPIA). (**B**) The mutation and CNA distribution of ACE2 in pan-cancer.

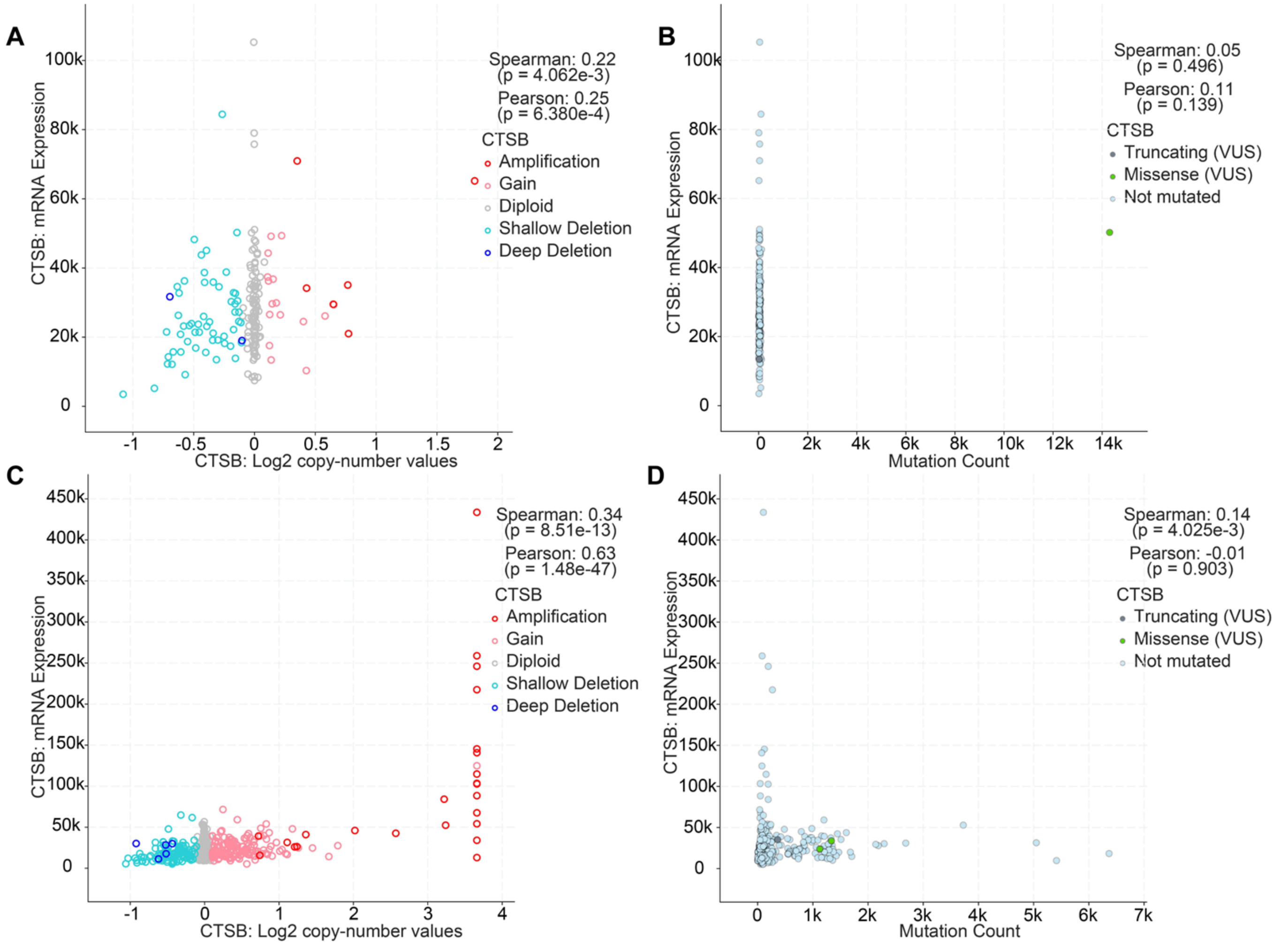

Supplementary Figure 2. The correlation analyses of Copy number values or Mutant count with mRNA expression for CTSB in PAAD (**A**, **B**) and STAD (**C**, **D**) (cBioportal). |R|>0.1, *P*<0.05 was regarded as having a correlation.

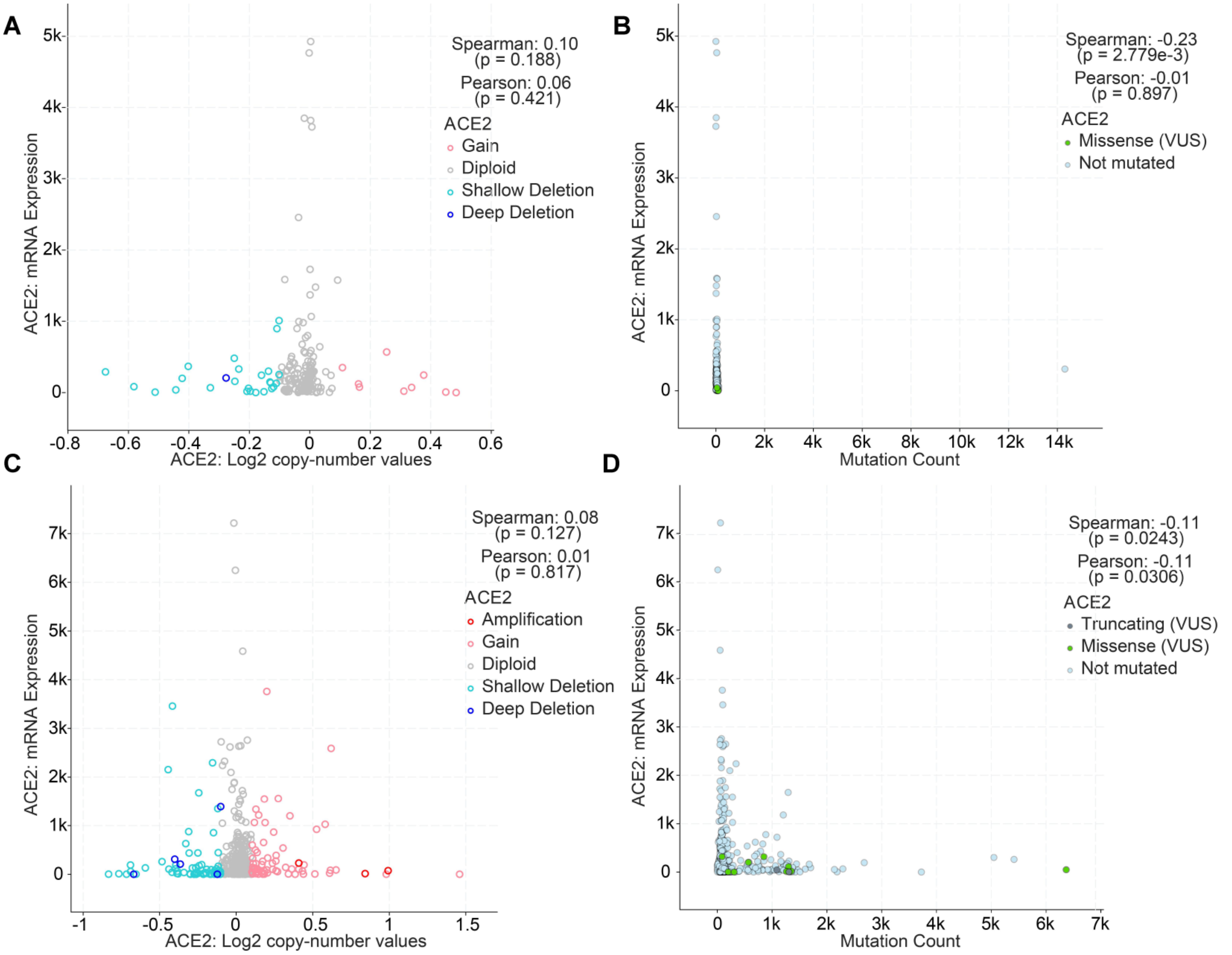

Supplementary Figure 3. The correlation analyses of Copy number values or Mutant count with mRNA expression for ACE2 in PAAD (**A**, **B**) and STAD (**C**, **D**) (cBioportal). |R|>0.1, *P*<0.05 was regarded as having a correlation.

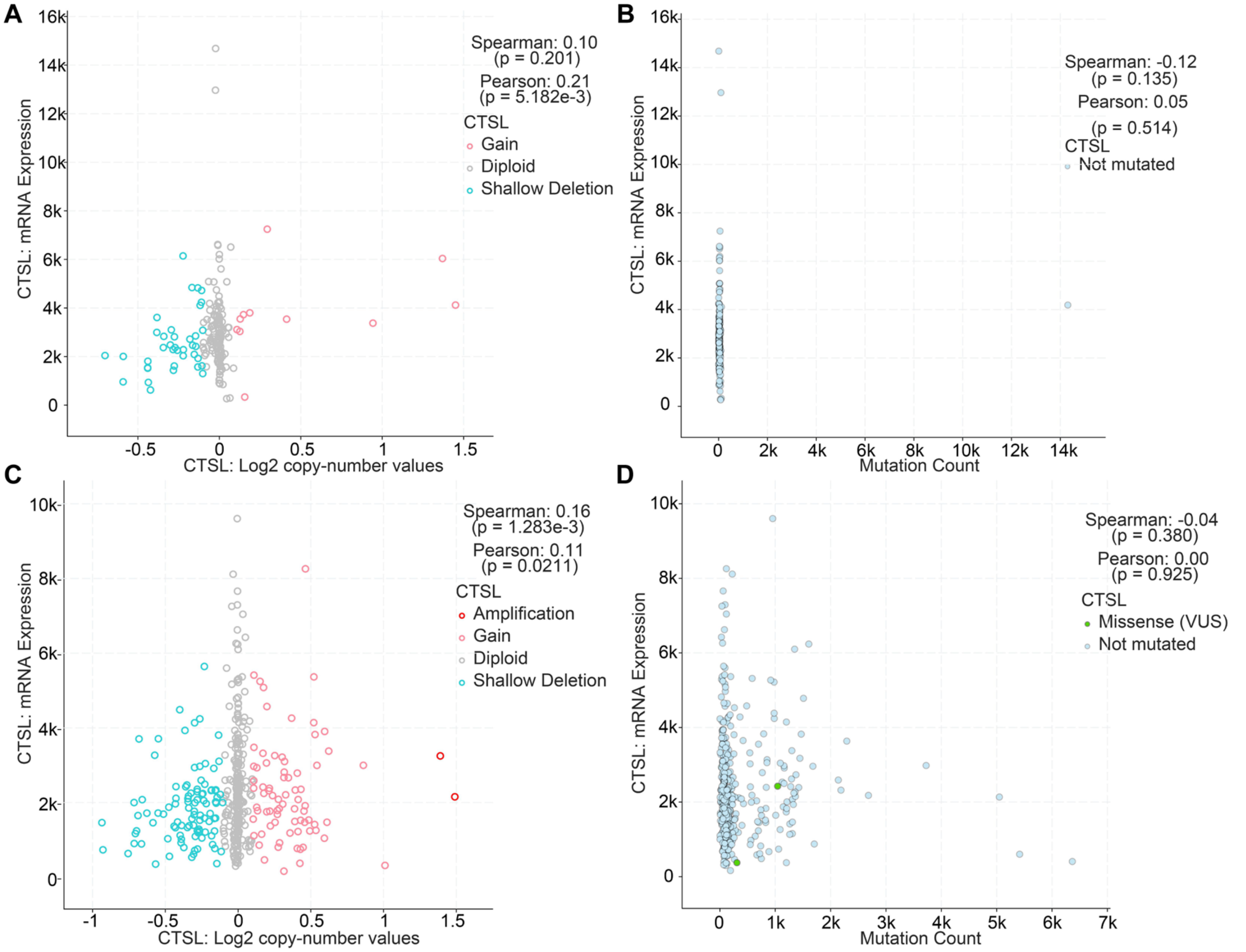

Supplementary Figure 4. The correlation analyses of Copy number values or Mutant count with mRNA expression for CTSL in PAAD (**A**, **B**) and STAD (**C**, **D**) (cBioportal). |R|>0.1, *P*<0.05 was regarded as having a correlation.

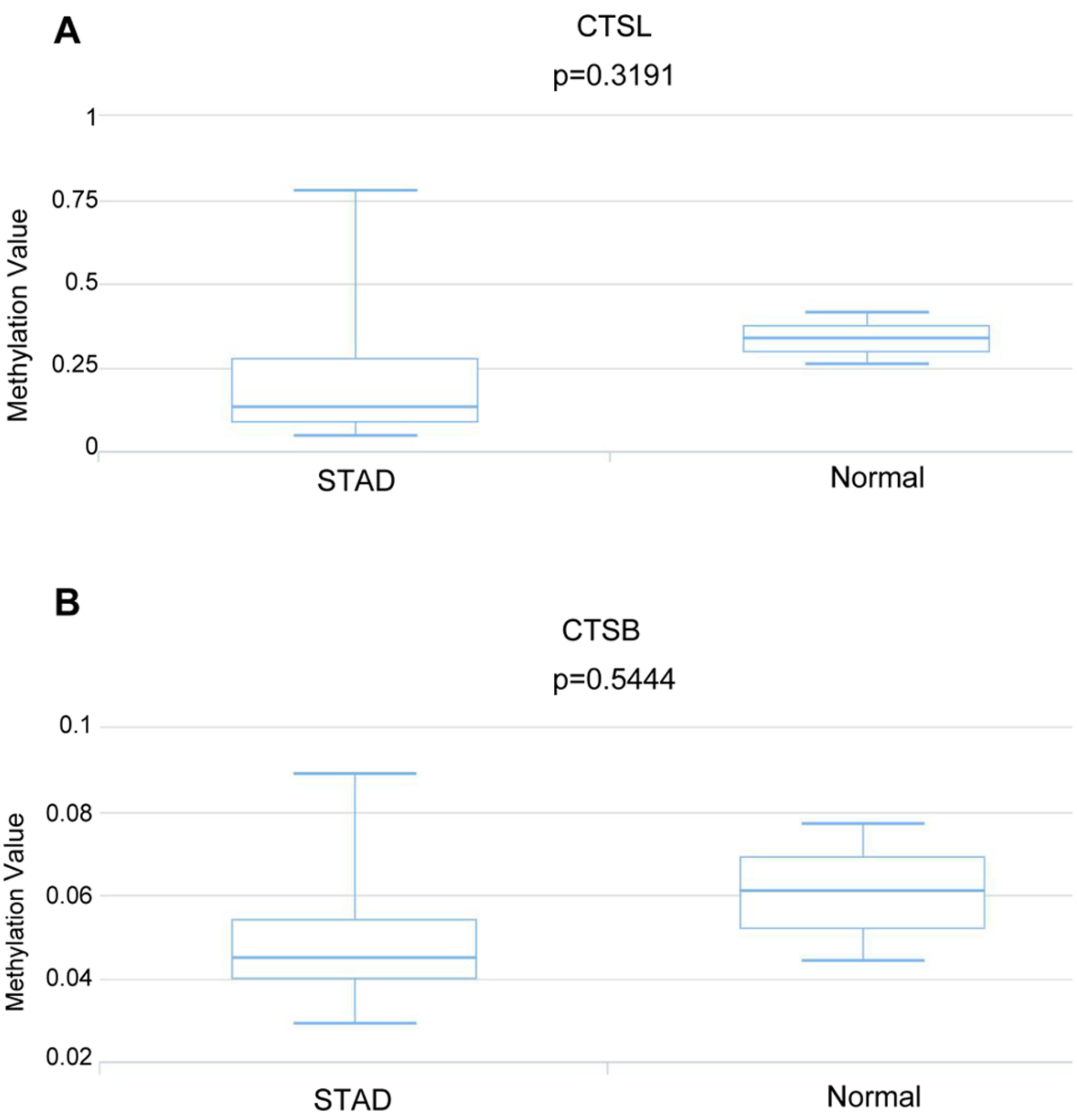

Supplementary Figure 5. The identification of DNA methylation for CTSL (**A**) and CTSB (**B**) in STAD (DiseaseMeth version 2.0). *P*<0.05 was regarded as significance.

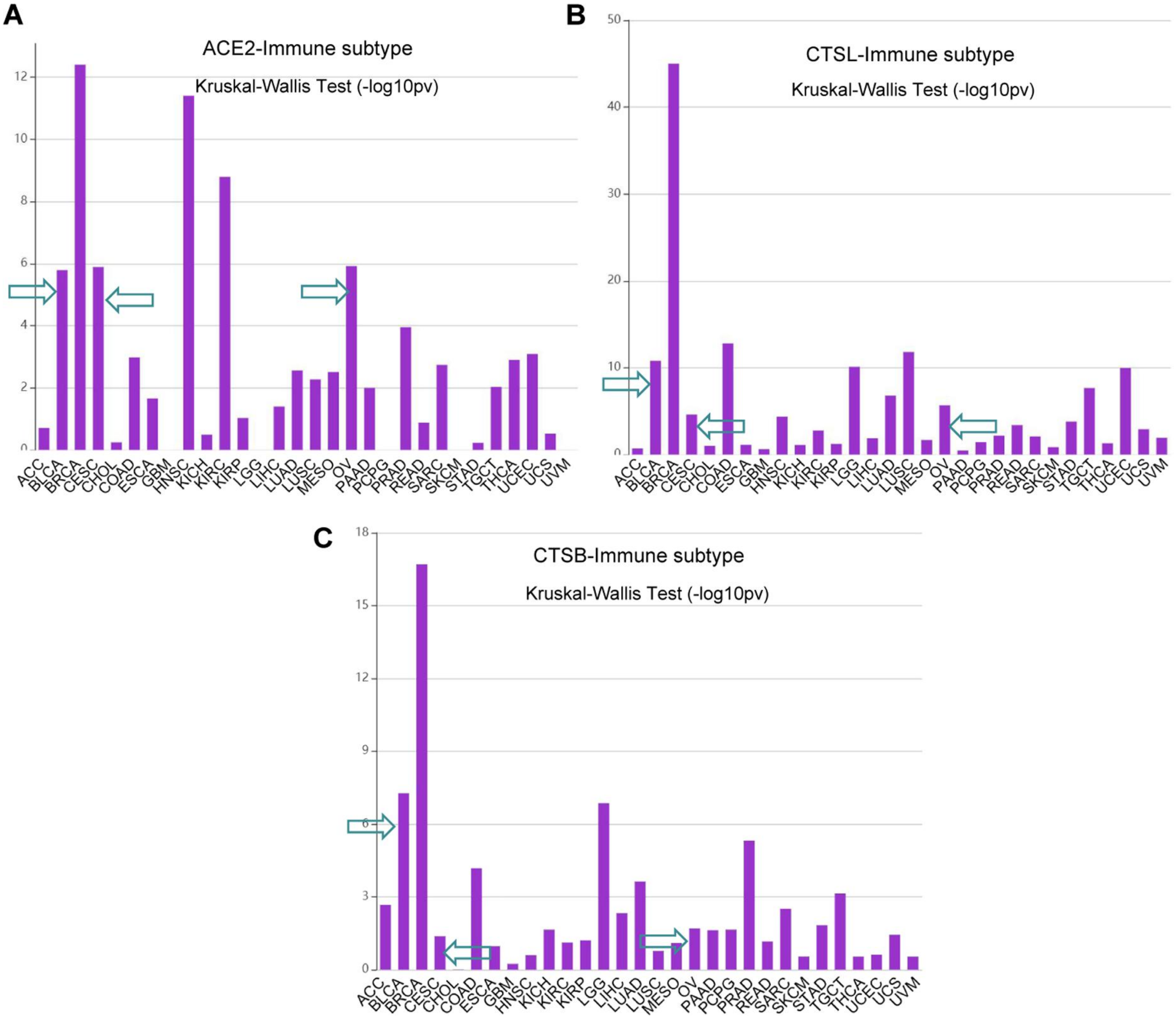

Supplementary Figure 6. The distribution analyses of immune subtypes including C1 (wound healing), C2 (IFN-gamma dominant), C3 (inflammatory), C4 (lymphocyte depleted), C5 (immunologically quiet) and C6 (TGF-b dominant) for ACE2 (**A**), CTSL (**B**) and CTSB (**C**) in 30 types cancers via TISIDB web tool. The green arrows represent the difference analyses of ACE2, CTSL, and CTSB among the immune subtypes of OV, CESC, and BLCA.

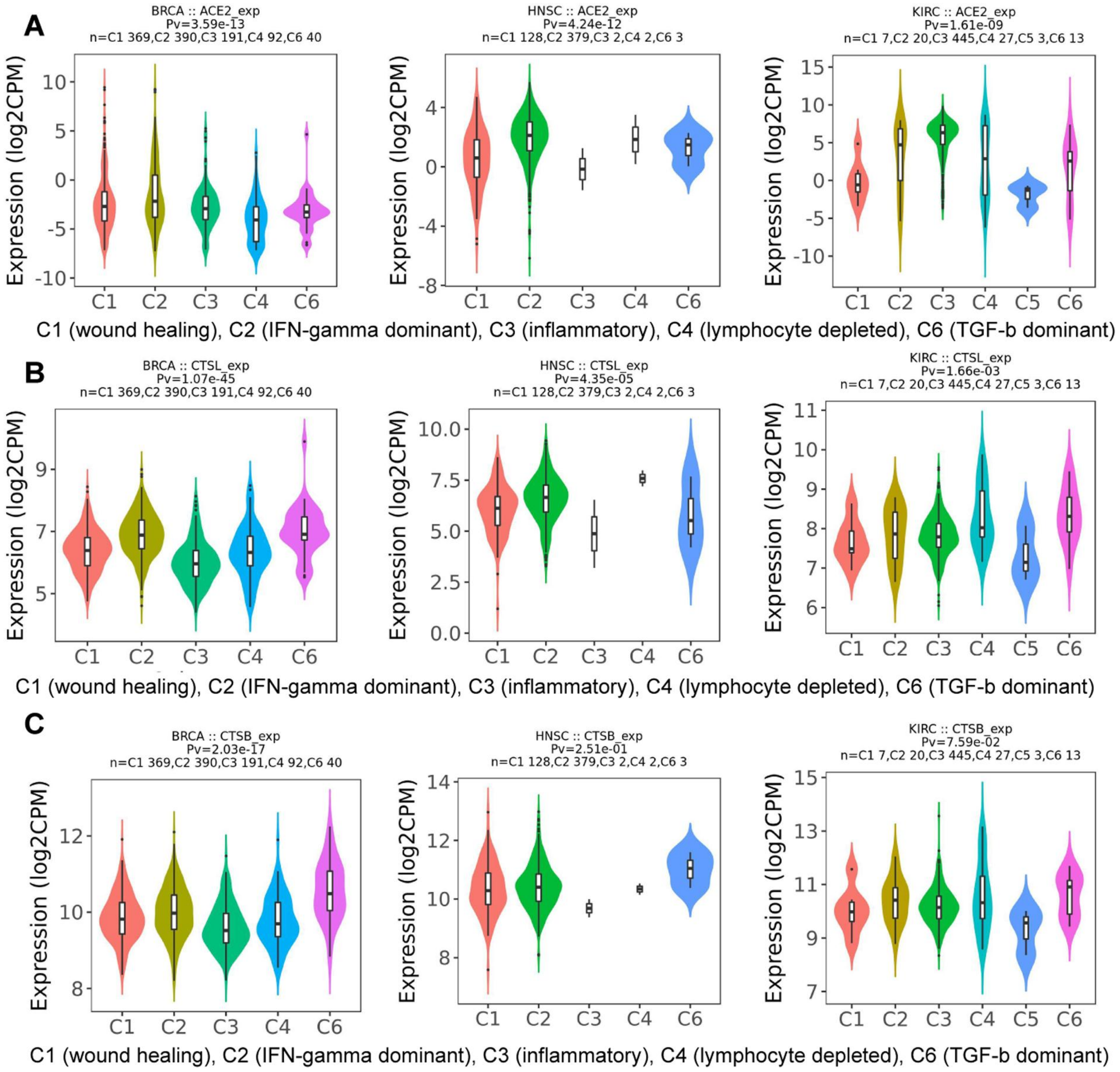

Supplementary Figure 7. The distribution graphs of ACE2 (**A**) and CTSL (**B**) and CTSB (**C**) in different immune subtypes in BRCA, HNSC, and KIRC (TISIDB). C1: wound healing; C2: IFN-gamma dominant; C3: inflammatory; C4: lymphocyte depleted; C5: immunologically quiet; C6: TGF-b dominant.

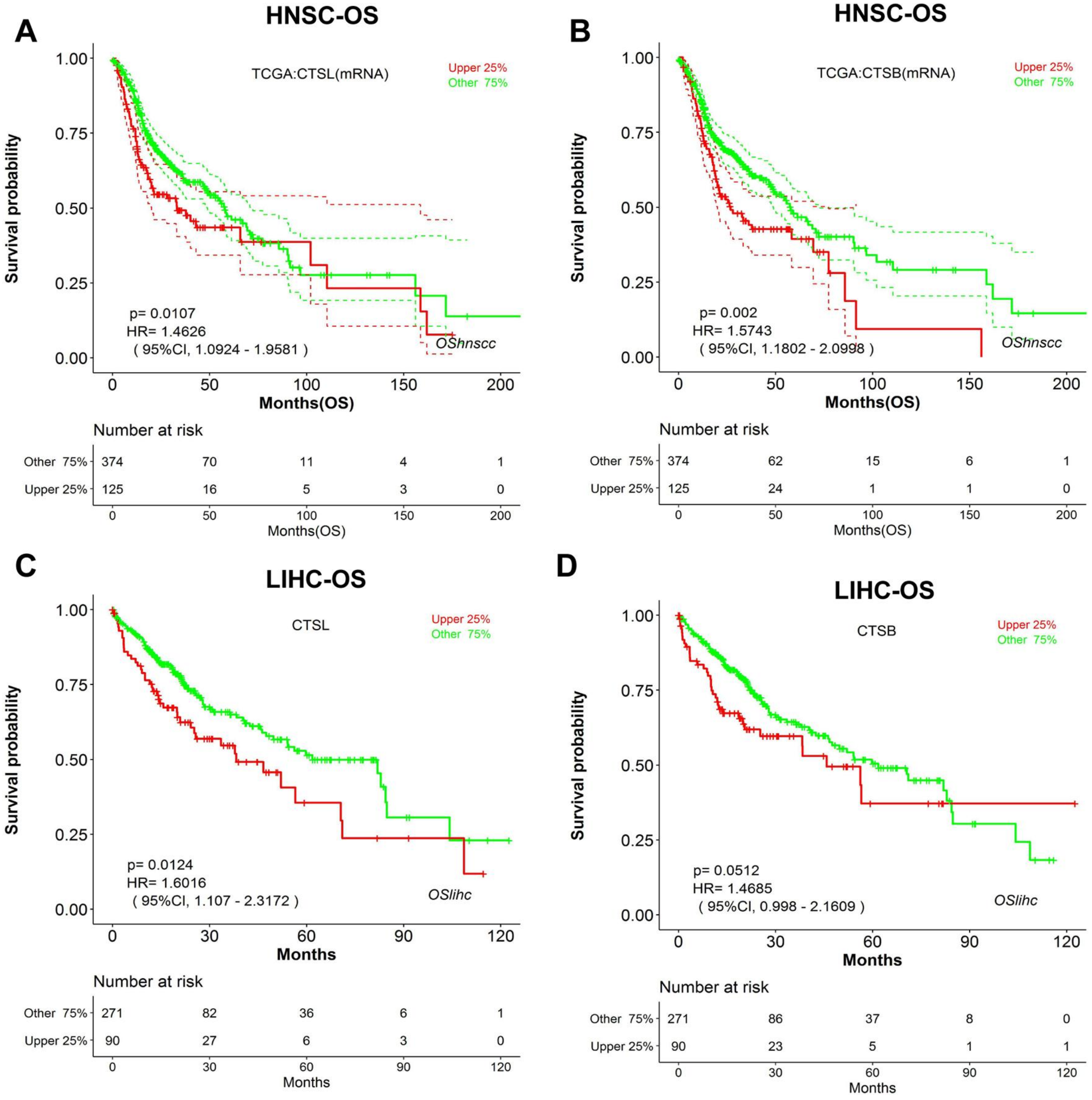

Supplementary Figure 8. The OS (Overall survival) analyses of CTSL (**A**) and CTSB (**B**) in HNSC utilizing the LOGpc web tool based on TCGA and GEO data. The OS analyses of CTSL(**C**) and CTSB (**D**) in LIHC utilizing the LOGpc web tool based on TCGA and GEO data.
