# Supplementary material for: Genomic, epigenomic, and immune subtype analysis of CTSL/B and SARS-CoV-2 receptor ACE2 in pan-cancer
Source: Aging (Albany NY). 2020 Nov 20;12(22):22370–89. doi: 10.18632/aging.104147 (PMC7746364; doi:10.18632/aging.104147)
Supplement: Supplementary Tables [file aging-12-104147-s002..pdf]

SUPPLEMENTARY TABLES

Supplementary Table 1. The distribution of CTSL in PAAD, STAD and their normal tissues.

Cancer types	Dataset	Tumor (Cases)	Normal (Cases)	Fold change	t-Test	*P*-value
PAAD	Badea Pancreas	Pancreatic Ductal Adenocarcinoma(39)	Pancreas(39)	3.124	8.078	1.40E-11
	Segara Pancreas	Pancreatic Carcinoma(11)	Pancreas(6)	3.233	3.966	7.32E-04
	lacobuzio-Donahue Pancreas	Pancreatic Adenocarinoma(12)	Pancreas(5)	2.811	4.173	0.004
	Grutzmann Pancreas	Pancreatic Ductal Adenocarcinoma(11)	Pancreas Duct(11)	1.791	1.667	0.056
	Pei Pancreas	Pancreatic Carcinoma(36)	Pancreas(16)	2.443	3.482	0.001
STAD	Chen Gastric	Gastric Intestinal Type Adenocarcinoma(64)	Gastric Mucosa(29)	2.424	13.942	9.87E-22
	Chen Gastric	Diffuse Gastric Adenocarcinoma(13)	Gastric Mucosa(29)	1.834	6.328	2.24E-06
	Chen Gastric	Gastric Mixed Adenocarcinoma(8)	Gastric Mucosa(29)	3.357	7.385	3.01E-05
	DErrico Gastric	Gastric Mixed Adenocarcinoma(4)	Gastric Mucosa(31)	2.847	16.872	3.40E-10
	DErrico Gastric	Gastric Intestinal Type Adenocarcinoma(26)	Gastric Mucosa(31)	3.837	10.142	2.67E-14
	DErrico Gastric	Diffuse Gastric Adenocarcinoma(6)	Gastric Mucosa(31)	2.378	4.596	1.00E-03
	Gui Gastric	Gastric Cancer(80)	Gastric Tissue(80)	1.594	6.047	5.15E-09
	Cho Gastric	Gastric Intestinal Type Adenocarinoma(20)	Gastric Tissue(19)	1.797	5.201	5.44E-06
	Cho Gastric	Diffuse Gastric Adenocarinoma(31)	Gastric Tissue(19)	1.776	6.056	1.05E-07
	Cho Gastric	Gastric Adenocarinoma(4)	Gastric Tissue(19)	2.073	2.696	3.30E-02
	Wang Gastric	Gastric Cancer(12)	Gastric Tissue(12) Gastric Mucosa(3)	1.948	3.419	1.00E-03

PAAD: Pancreatic adenocarcinoma; STAD: Stomach Adenocarcinoma.

Supplementary Table 2. The distribution of CTSB in PAAD, STAD and their normal tissues.

Cancer types	Dataset	Tumor(Cases)	Normal(Cases)	Fold change	t-Test	*P*-value
PAAD	Logsdon Pancreas	Pancreatic Adenocarcinoma(10)	Pancreas(5)	1.614	2.225	0.022
	Badea Pancreas	Pancreatic Dutal Adenocarcinoma(39)	Pancreas(39)	1.975	6.077	3.70E-08
	lacobuzio-Donahue Pancreas 2	Pancreatic Adenocarcinoma(12)	Pancreas(5)	1.797	1.727	0.069
	Pei Pancreas	Pancreastic Carcinoma(36)	Pancreas(16)	1.514	2.525	0.009
STAD	Chen Gastric	Gastric Mixed Adenocarcinoma(8)	Gastric Mucosa(29)	2.844	11.259	4.29E-09
	Chen Gastric	Gastric Intestinal Type Adenocarcinoma(67)	Gastric Mucosa(29)	2.717	14.424	6.38E-22
	Chen Gastric	Diffuse Gastric Adenocarcinoma(13)	Gastric Mucosa(29)	2.538	5.275	5.12E-05
	DErrico Gastric	Gastric Intestinal Type Adenocarcinoma(26)	Gastric Mucosa(31)	2.357	8.821	7.30E-11
	DErrico Gastric	Diffuse Gastric Adenocarcinoma(6)	Gastric Mucosa(31)	1.887	4.323	3.00E-03
	DErrico Gastric	Gastric Mixed Adenocarcinoma(4)	Gastric Mucosa(31)	2.772	3.866	1.40E-02
	Wang Gastric	Gastric Cancer(12)	Gastric Mucosa(12)<br>Gastric Tissue(3)	2.273	3.988	5.18E-04
	Cho Gastric	Diffuse Gastric Adenocarcinoma(31)	Gastric Tissue(19)	1.793	3.673	3.07E-04

PAAD: Pancreatic adenocarcinoma; STAD: Stomach Adenocarcinoma.

Supplementary Table 3. The CpG methylation probes located in promoters of CTSL/B and ACE2.

Gene Symbol	CpG	Group	Relation To Island
CTSL	cg11154542	TSS1500	N_Shore
CTSL	cg11306701	5'UTR	Island
CTSL	cg13985445	TSS1500	N_Shore
CTSL	cg14236855	5'UTR;1stExon	Island
CTSL	cg14243623	5'UTR;1stExon	Island
CTSL	cg14557714	5'UTR	Island
CTSL	cg15242570	5'UTR;1stExon	Island
CTSB	cg00057317	5'UTR	Island
CTSB	cg00276098	TSS1500	S_Shore
CTSB	cg02637136	5'UTR;1stExon	Island
CTSB	cg04315434	TSS1500	Island
CTSB	cg08012294	5'UTR	N_Shore
CTSB	cg16624891	5'UTR	OpenSea
CTSB	cg17019285	5'UTR	Island
CTSB	cg18787975	TSS1500	S_Shore
CTSB	cg20539307	5'UTR	OpenSea
CTSB	cg21281001	5'UTR	Island
CTSB	cg21756720	5'UTR	N_Shore
CTSB	cg21919729	5'UTR	OpenSea
CTSB	cg22139271	5'UTR;1stExon	Island
CTSB	cg26407100	TSS1500	Island
ACE2	cg05748796	5'UTR	OpenSea
ACE2	cg08559914	TSS200	OpenSea
ACE2	cg16734967	5'UTR;1stExon	OpenSea
ACE2	cg18458833	TSS1500	OpenSea
ACE2	cg18877734	TSS1500	OpenSea
ACE2	cg21598868	TSS1500	OpenSea

Supplementary Table 4. The correlation analysis of DNA methylation level of CTSL to its gene expression in pan-cancer.

Disease	GeneSymbol	Pearson R	Pearson p-value
BLCA	CTSL	-0.00312611	0.948826
BRCA	CTSL	-0.027521	0.418322
CESC	CTSL	-0.366557	0
CHOL	CTSL	-0.239902	0.112444
COAD	CTSL	-0.0123726	0.82659
ESCA	CTSL	-0.252473	0.000863984
GBM	CTSL	-0.255165	0.04186
HNSC	CTSL	-0.301539	0
KIRC	CTSL	-0.0301101	0.578949
KIRP	CTSL	-0.0844174	0.146702
LIHC	CTSL	-0.0159312	0.74655
LUAD	CTSL	-0.0655713	0.153184
LUSC	CTSL	-0.148525	0.00384861
PAAD	CTSL	-0.0665734	0.371896
PCPG	CTSL	-0.0782841	0.289514
PRAD	CTSL	0.0319835	0.462059
READ	CTSL	0.0645515	0.521309
SARC	CTSL	0.0854136	0.167247
SKCM	CTSL	-0.340934	0
STAD	CTSL	-0.0153432	0.778991
THCA	CTSL	0.11606	0.00601102
THYM	CTSL	-0.142602	0.118683
UCEC	CTSL	0.0546759	0.240321

BLCA: Bladder Urothelial Carcinoma; BRCA: Breast invasive carcinoma; CESC: Cervical squamous cell carcinoma and endocervical adenocarcinoma; CHOL: Cholangio carcinoma; COAD: Colon adenocarcinoma; ESCA: Esophageal carcinoma; GBM: Glioblastoma multiforme; HNSC: Head and Neck squamous cell carcinoma; KICH: Kidney Chromophobe; KIRC: Kidney renal clear cell carcinoma; KIRP:Kidney renal papillary cell carcinoma; LIHC: Liver hepatocellular carcinoma; LMS: Leiomyosarcoma; LUAD: Lung: adenocarcinoma; LUSC: Lung squamous cell carcinoma; PAAD: Pancreatic adenocarcinoma; PCPG: Pheochromocytoma and Paraganglioma; PRAD: Prostate adenocarcinoma; READ: Rectum adenocarcinoma; SARC: Sarcoma; SKCM: Skin Cutaneous Melanoma; STAD: Stomach Adenocarcinoma; THCA: Thyroid carcinoma; THYM: Thymoma; UCEC: Uterine Corpus Endometrial Carcinoma; $0.1<|R|<0.3$ and $P<0.05$ was regarded as weak correlation; $0.3<|R|<0.5$ and $P<0.05$ was regarded as moderate correlation; $|R|>0.5$ and $P<0.05$ was regarded as strong correlation.

Supplementary Table 5. The correlation analysis of DNA methylation level of ACE2 to its gene expression in pan-cancer.

Disease	GeneSymbol	Pearson R	Pearson p value
BLCA	ACE2	-0.22944	0.000001803
BRCA	ACE2	-0.150097	9.0638E-06
CESC	ACE2	0.0420417	0.461514
CHOL	ACE2	0.000887264	0.995385
COAD	ACE2	-0.594137	1.5447e-31
ESCA	ACE2	-0.123937	0.106303
GBM	ACE2	-0.00669388	0.958133
HNSC	ACE2	-0.114473	0.00885114
KIRC	ACE2	-0.0672159	0.215015
KIRP	ACE2	0.104754	0.0714444
LIHC	ACE2	-0.365046	0
LUAD	ACE2	-0.208215	4.6257E-06
LUSC	ACE2	-0.0820471	0.111732
PAAD	ACE2	0.214959	0.00356698
PCPG	ACE2	0.0938661	0.203775
PRAD	ACE2	-0.00895685	0.836858
READ	ACE2	-0.51804	2.89e-8
SARC	ACE2	0.100805	0.102859
SKCM	ACE2	0.0787119	0.0893085
STAD	ACE2	-0.0777361	0.154476
THCA	ACE2	0.156607	0.000201307
THYM	ACE2	0.0975874	0.286938

BLCA: Bladder Urothelial Carcinoma; BRCA: Breast invasive carcinoma; CESC: Cervical squamous cell carcinoma and endocervical adenocarcinoma; CHOL: Cholangio carcinoma; COAD: Colon adenocarcinoma; ESCA: Esophageal carcinoma; GBM: Glioblastoma multiforme; HNSC: Head and Neck squamous cell carcinoma; KICH: Kidney Chromophobe; KIRC: Kidney renal clear cell carcinoma; KIRP:Kidney renal papillary cell carcinoma; LIHC: Liver hepatocellular carcinoma; LMS: Leiomyosarcoma; LUAD: Lung: adenocarcinoma; LUSC: Lung squamous cell carcinoma; PAAD: Pancreatic adenocarcinoma; PCPG: Pheochromocytoma and Paraganglioma; PRAD: Prostate adenocarcinoma; READ: Rectum adenocar -cinoma; SARC: Sarcoma; SKCM: Skin Cutaneous Melanoma; STAD: Stomach Adenocarcinoma; THCA: Thyroid carcinoma;THYM: Thymoma; UCEC: Uterine Corpus Endometrial Carcinoma; $0.1<|R|<0.3$ and $P<0.05$ was regarded as weak correlation; $0.3<|R|<0.5$ and $P<0.05$ was regarded as moderate correlation; $|R|>0.5$ and $P<0.05$ was regarded as strong correlation.

Supplementary Table 6. The correlation analysis of DNA methylation level of CTSB to its gene expression in pan-cancer.

Disease	GeneSymbol	Pearson R	Pearson p value
BLCA	CTSB	-0.0862408	0.0760846
BRCA	CTSB	-0.101124	0.00287371
CESC	CTSB	0.0331376	0.561709
CHOL	CTSB	-0.334951	0.0245063
COAD	CTSB	-0.0965971	0.0864639
ESCA	CTSB	-0.283268	0.000173876
GBM	CTSB	0.00697352	0.956386
HNSC	CTSB	-0.0901373	0.0395278
KIRC	CTSB	0.114073	0.0349671
KIRP	CTSB	-0.125537	0.0305488
LIHC	CTSB	-0.183504	0.000173767
LUAD	CTSB	-0.0666532	0.146504
LUSC	CTSB	-0.161446	0.00166104
PAAD	CTSB	-0.187391	0.0113059
PCPG	CTSB	-0.114122	0.121919
PRAD	CTSB	-0.284738	0
READ	CTSB	-0.239448	0.0158798
SARC	CTSB	-0.0538639	0.384302
SKCM	CTSB	-0.194021	2.42374E-05
STAD	CTSB	-0.118964	0.028996
THCA	CTSB	-0.0641417	0.129853
THYM	CTSB	0.00115651	0.989955
UCEC	CTSB	0.11232	0.0156076

BLCA: Bladder Urothelial Carcinoma; BRCA: Breast invasive carcinoma; CESC: Cervical squamous cell carcinoma and endocervical adenocarcinoma; CHOL: Cholangio carcinoma; COAD: Colon adenocarcinoma; ESCA: Esophageal carcinoma; GBM: Glioblastoma multiforme; HNSC: Head and Neck squamous cell carcinoma; KICH: Kidney Chromophobe; KIRC: Kidney renal clear cell carcinoma; KIRP:Kidney renal papillary cell carcinoma; LIHC: Liver hepatocellular carcinoma; LMS: Leiomyosarcoma; LUAD: Lung: adenocarcinoma; LUSC: Lung squamous cell carcinoma; PAAD: Pancreatic adenocarcinoma; PCPG: Pheochromocytoma and Paraganglioma; PRAD: Prostate adenocarcinoma; READ: Rectum adenocarcinoma; SARC: Sarcoma; SKCM: Skin Cutaneous Melanoma; STAD: Stomach Adenocarcinoma; THCA: Thyroid carcinoma; THYM: Thymoma; UCEC: Uterine Corpus Endometrial Carcinoma; $0.1<|R|<0.3$ and $P<0.05$ was regarded as weak correlation; $0.3<|R|<0.5$ and $P<0.05$ was regarded as moderate correlation; $|R|>0.5$ and $P<0.05$ was regarded as strong correlation.
